# Outlines of a New System of Philosophy, Being a View of the System of Sciential Medicine: Or Medicine (and All Human Knowledge) as Proveable as Geometry

**Published:** 1835-01-01

**Authors:** 


*Outlines of a New System of Philosophy, being a View of the System of Sciential Medicine: or Medicine (and all Human Knowledge) as proveable as Geometry.* By THOMAS EDEN, M.R.C.S. London. 1834. 12mo. pp. 262.

AMONG the oddities which foreigners have remarked in our national character, few seem to have struck them more than the propensity of every true-born Briton to lay wagers; and they mention with surprise that a sum sufficient to purchase a French dukedom is often staked on an event, which, however interesting, must be allowed to be of minor importance; such as a race between two maggots, or the like. The Prince of Wales and Mr. Fox once laid a bet as to which should make the worst pun; and we cannot help thinking that the queer nothingness, the expanded zero, in the shape of a book, with which Mr. Eden has favoured us, owes its existence to a similar agreement; the bet was probably to be won by him who should write the most foolish book of the season; and impartiality obliges us to confess that our author has immeasurably distanced every competitor—he wins at a canter. The grand gist of the book consists in a perpetual talk about "science," a word which occurs half a dozen times in a page; though the thing is as much like a scientific treatise as Bombastes Furioso is like a great general, or the Sir Frederick Fineer of a set of Twelfth Night characters resembles a man of fashion.

There is a very pleasant section in the book, where Mr. Eden gives an abstract of the unanimous opinion of the profession about himself and his science; but we should rather take it to be a prophetic glimpse of a hundred reviews of the book, in case it should be our author's destiny to be immortalized in so many.

"Ill thinkers who look not to truth have ever thus referred new doctrines to their preconceived notions, and have tolerated or condemned, by their mere pre-judgings; such a decider has human nature ever been, most censuring that which least accords with error, and most condemning that truth which is the most corrective. And soon as the name of sciential medicine had been uttered, before one doctrine had been whispered, sudden condemnation was past upon the science and myself; from every person to whom the name had gone sudden censure was received; condemning epithets soon poured thickly in; 'stuff;' 'all moonshine;' 'silly notions;' 'mistaken notions altogether;' 'nonsense;' 'arrant nonsense no doubt;' 'doctrines founded, no doubt, on some fundamental error;'

'great folly;' 'doctrines which he dare not openly advance;' 'which he dare not give a glimmering explanation of;' 'quackery;' 'silly doctrines which I will undertake to refute and expose in a moment;' 'a silly man;' 'led astray by some error;' 'whose head is full of mistaken notions;' 'a well-meaning young man that I am sorry for;' 'that I pity;' 'a person who ought to be put under the care of his friends;' 'a poor fellow who has no friends to take care of him;' 'a junior practitioner led astray by the ardour of his feelings;' 'an enthusiast;' 'a violent enthusiast:' 'a visionary;' 'a great visionary;' 'a fool;' 'a prating fool;' 'a contemptible fool:' these are some of the many epithets which I have heard of; and there is no scoffing epithet of common contempt, nor any sneering epithet of derision however high, which 'my dearly beloved brethren' of the profession have not, by their whisperings and mutterings, already thrown upon sciential medicine and myself." (P. 24.)

This is a very fair parody, our readers must allow, on the manner of those nervous beings who meet misfortune half-way, and anticipate what the critics will say of them; but our author abounds in these imitations. For instance, it is very common for the unreadable to cry out, that as Milton was neglected, so are they; just as a pig-stealer might say "Aristides was banished, so am I:" and thus our author exults in the neglect of "sciential" medicine.

"Thus you see sciential medicine has already on its head many of those leaves without a crown of which no corrective truth ever went triumphantly forth: the science is already under the invigorating auspices of that same patronizing power which has ushered into the world and attended the infancy of the greatest discoveries which philosophy ever gave birth to." (P. 26.)

If we wished to give our readers the best notion of this ebullition of nonsense, we should call it a very grotesque parody on logical and metaphysical writers. Here is a delicious bit of definition.

"A science, or a portion of knowledge, or a truth, of the individual, or of the few, or of the many, or of the multitude, is of, or belonging to, science, knowledge, truth." (P. 160, *note.*)

Or take the first and second positions of sciential medicine.

"Whatever individual thing does, according to your individual power to know, exist, does exist according to truth; and whatever individual thing does not, according to your individual power to know, exist, does not exist, according to truth." (P. 107.)

"'Whatever individual thing does, according to your individual power to know, exists in a specific time, place, combination, &c., does exist in that time, place, combination, &c., according to truth; and whatever individual thing does not, according to your individual power to know, liably exist in a specific time, place, combination, &c., does not exist in that time, place, combination, &c., according to truth." (P. 156.)

Or this agreeable specimen of a 30-page dialogue between those obstinate persons, Messrs. Pro and Con.

" *Con.* Then, I appeal in the same manner to a person (my teacher) who knows what I do not know.

" *Pro.* Then I give (distributing names differently) the same reply.

" 132. *Con.* According to my power to know! according to his power to know! according to troy! according to avoirdupois! I know the decisions are different, but truth must be something independent of us, and of our decisions; the matter is for men to get at the truth, not for them to decide anyhow, as you teach.

" *Pro.* Sciential medicine teaches but one manner of decision. Do not delude yourself by such words as 'men,' and 'get at the truth.' By 'men' you mean, men one after another, that is, some men first. There is no truth, but what some men are at; for truth is always a mental affection; it never is 'independent.'

" 133. *Con.* But some mental affections are right, and some are wrong.

" *Pro.* And you have undertaken to show that the mental affections, obtained by the rules of sciential medicine, are wrong." (P. 117.)

Our readers will allow that in this style Mr. Eden is unrivalled, and does his splutter more completely than any one within the memory of man; he should keep to this, and never attempt a real book; "motley is his only wear." Our author is on the look out for ten thousand antagonists: he imagines that the "professors of acephalous medicine," i. e. all the doctors, great and small, will be eager to refute his babble, and will pester him with unpaid letters. But let him fear nothing; every one will accept our explanation of the nature and origin of his essay; and the doctors will no more write to Mr. Eden to shew that his fun is not philosophy, than they do to clown and pantaloon, to tell them that their complexions are not genuine.

